# Contribution of Medical Wards Contamination to Wound Infection among Patients Attending Ruhengeri Referral Hospital

**DOI:** 10.1155/2021/7838763

**Published:** 2021-10-11

**Authors:** Emmanuel Munyeshyaka, Parfait Cyuzuzo, Callixte Yadufashije, John Karemera

**Affiliations:** ^1^Biomedical Laboratory Sciences Department, INES-Ruhengeri Institute of Applied Sciences, Ruhengeri, Musanze, Rwanda; ^2^Microbiology Unity, Rwanda Forensic Laboratory, Kigali, Rwanda

## Abstract

Nosocomial infections or hospital-acquired infections are infections that potentially occur in the patients under medical care. These infections are often caused by multidrug-resistant pathogens acquired via improper antibiotic use, not following infection control and prevention procedures. The main objective of this study was to investigate the contribution of medical wards contamination to wound infection and antibiotics susceptibility patterns at Ruhengeri Referral Hospital, Musanze district, Rwanda. This was a cross-sectional study where a total of 61 samples including air sampling to evaluate the contamination by airborne bacteria, working surface, equipment, and patients' surgical wounds swabs were collected in intensive care unit (ICU), pediatrics, and surgery departments. Culture, Gram stain, and biochemical tests were performed for microbiological isolation and identification. Antibiotic susceptibility testing was performed using the Kirby–Bauer disc diffusion method. Statistical Package for Social Science (SPSS) version 22 was used for data analysis. Gram-negative bacteria were frequently from surgery, pediatric, and ICU with 68.8%, 63.9%, and 31.1%, respectively, while Gram-positive isolates were 37.7% in surgery, 32.9% in pediatric, and 18.0% in ICU. There was a statistically significant association with *E. coli* and swabbed materials and surgical wound sites (*x*^2^ = 10.0253, *P* value = 0.018). All bacterial contaminants were sensitive to clindamycin and erythromycin. *Pseudomonas aeruginosa*, *E. coli*, and *S. aureus* were resistant to nitrofurantoin. Hospital environment could be a contributing factor to surgical wound site infections. Hospitals should apply preventive measures in the hospital environment surrounding wound surgery patients to prevent wound infections during hospital stay.

## 1. Introduction

Nosocomial infections, also known as healthcare-associated infections (HAI), are infections acquired during the process of receiving healthcare that was not present during the time of admission [1]. Hospital-acquired infections are a major cause of morbidity and mortality worldwide. Infections caused by multidrug-resistant (MDR) bacteria are a worrying healthcare problem and a daily challenge for the clinician dealing with critically ill patients [2]. Additionally, they are often caused by breaches of infection control practice and procedures, unclean and nonsterile environmental surfaces, and ill employees who can provide opportunity for these pathogens for surviving to cause infections. Thus, healthcare facilities can be dangerous places for the acquisition of infections [3]. Bacteria were reported to be the most common pathogens responsible for nosocomial infections that account for 90% of these cases [4]. Mainly, some bacteria belong to natural flora of the patients but cause infection only when the immune system of the patient becomes prone to infections [2].

However, nosocomial infections can cause severe pneumonia, infections of the urinary tract, bloodstream, and infections to other parts of the body [5]. Though, several bacteria display antimicrobial resistance, which can complicate treatment [4]. According to the CDC report, the most common pathogens from medical wards contamination causing nosocomial infections are *Staphylococcus aureus*, *Pseudomonas aeruginosa*, and *Escherichia coli* [6]. Furthermore, some of the common nosocomial infections are urinary tract infections, respiratory pneumonia, surgical site wound infections, bacteremia, gastrointestinal, and skin infections [7]. *Clostridium difficile* was reported as the chief cause of nosocomial diarrhea in the US and Europe [8]. Whereas, methicillin-resistant *Staphylococcus aureus* (MRSA) are resistant to certain antibiotics and may be acquired during hospitalization [9]. Furthermore, nosocomial infections are not just limited to bacteria; certain fungi, including *Candida albicans* and *Aspergillus*, and also, viruses, such as respiratory syncytial virus (RSV) and influenza, have also been implicated in a number of hospital-acquired infections but to the smaller extent [10].

Nosocomial infections due to medical wards contamination occur worldwide both in developed and developing countries; the infection accounts for 7% in developed and 10% in developing countries [1]. According to estimate report of CDC, in the United States, approximately 1.7 million of hospital-acquired infections from all types of microorganisms including bacteria and fungi combined contribute to 99,000 deaths each year [11]. Meanwhile, a total of 8.9 million healthcare-associated infections were estimated to occur annually in European hospitals, and long-term care facilities and categories of Gram-negative infections are estimated to account for two-thirds of the 25,000 deaths each year [12]. Within available data, the incidence of nosocomial infections in sub-Saharan Africa ranges 2–49% in the patients hospitalized in intensive care units (ICU) with the highest rate ranging 21.2–35.6% [13]. Furthermore, nosocomial infections are estimated to make patients stay in the hospital 4-5 additional days [3]. Around 2004-2005, in France, about 9,000 people died each year with a nosocomial infection, of which about 4,200 would have survived without this infection [14]. The aim of this study was to determine contribution of medical wards contamination to wound infection among patients attending Ruhengeri Referral Hospital. The clinics and hospitals in Rwanda are crowded, creating atmosphere for hospital-acquired infections, and yet, few studies have been conducted related to medical wards contamination.

## 2. Materials and Methods

### 2.1. Study Area and Design

Study was conducted at Ruhengeri Referral Hospital located in Muhoza sector, Musanze district, Northern Province of Rwanda. This was an analytical cross-sectional study and was conducted from January to March 2019. Swab samples were taken from surface and patients who had developed wound infection and samples of airborne contamination within three different units: surgery, intensive care unit, and pediatric. The collected samples were analyzed in INES-Ruhengeri Clinical Microbiology Laboratory.

### 2.2. Study Population and Sample Size

The target population of this study was patients with wounds or any kind of wound admitted in surgery, ICU, and pediatric departments at Ruhengeri Referral Hospital. A total of 61 swab samples including 23 wound swabs, 12 blood agar plates for airborne contamination, and 26 swabs from surface samples were collected. All samples were adequately collected for the cultured and antibiogram test for analysis of identified bacteria at INES-Ruhengeri Clinical Microbiology Laboratory.

### 2.3. Swab Samples Collection

Swab samples from patients who had developed wound infection were collected aseptically and gently to avoid contamination of the specimens with normal microbial flora of the skin. The swab specimens were collected before dressing and administration of antibiotic therapy. The surface sample swabs (blanket or bed sheet, doors, wall near patients, and hygienic material: plastic wash basins or backet) were also collected by means of moistened swabs in sterile saline solution by bearing on the surface of each sample. All collected swabs were immediately inserted into Amies transport medium and labeled. Therefore, specimens were kept in a thermoflask containing ice and transported immediately to INES-Ruhengeri Clinical Microbiology Laboratory for bacteriological analysis. Air sampling plates were directly incubated aerobically at 35–37°C for 18–24 hrs.

### 2.4. Sample of Airborne Biocontamination

The sedimentation method was used for sampling of airborne biocontamination in the selected medical wards. Two series of samples were collected in the early morning before wound dressing once a week in the patient's rooms and during wound dressing on a certain day for a particular patient by the sedimentation method with blood agar plates placed at one meter on the ground at the head of the bed and left open for 2 hours. Blood agar growth media used for these samples were aerobically incubated at 37°C for 48 h. Therefore, positive plates with visible colonies were identified, and an antibiogram was performed by the Kirby–Bauer method.

### 2.5. Laboratory Analysis

#### 2.5.1. Bacterial Isolation and Identification

The collected specimen swabs were inoculated by using the streaking technique to expose bacteria in a good growing medium of blood agar and MacConkey agar Petri dishes. The plates were aerobically incubated at 37°C for 24 hours. Then, the growing colonies were morphologically identified and followed by Gram stain. Different biochemical tests were also performed to differentiate and confirm bacteria species. The catalase test was performed to differentiate Gram-positive cocci such as *Streptococcus* and *staphylococcus* species followed by the free coagulase test used to distinguish *Staphylococcus* coagulase-negative and coagulase-positive. For the identification of Gram-negative bacteria isolates, different biochemical culture media were used to test different biochemical parameters. Simmons citrate agar was used for the differentiation of microorganisms on the basis of citrate utilization. Sulfide indole and motility (SIM) was used to test motile Gram-negative bacilli bacteria with swimming away from a stab mark and hydrogen sulfide production. However, bacteria ability to produce enzyme tryptophanase was confirmed by adding 0.5 ml of Kovac's after 24 hours of incubation. Furthermore, Kligler iron agar (KIA) permitted differentiation of Gram-negative bacilli by their ability to ferment glucose or lactose was also used.

#### 2.5.2. Antibiotic Sensitivity Test

The Kirby–Bauer disc diffusion method was used to test the in vitro susceptibility of the identified isolates. The identified bacteria were *Staphylococcus aureus*, *Escherichia coli*, *Pseudomonas aeruginosa*, *Proteus mirabilis*, and *Enterobacter cloacae.* Antibiotics used were gentamicin (GM) 10 mcg, ciprofloxacin (CIP) 5 mcg, clindamycin (DA), oxacilin (OX) 10 *μ*g, nitrofurantoin (F) 50 mcg, and cefuroxime (CXM) 25 *μ*g. Resistance and sensitivity were considered by referring to the standard of each antibiotic.

The surface of Mueller-Hinton agar in a Petri dish was evenly inoculated with the suspension by using a sterile swab. Antibiotic discs were then put on the surface of the inoculated plate and incubated between 18 and 24 hours at 37°C. Therefore, the inhibition zones were then measured, and the interpreted disc diffusion method of bacteria for clear inhibition zones to determine if bacteria were resistant or sensitive on different antibiotic disc was performed.

### 2.6. Statistical Analysis

Data entry and analysis were performed using Statistical Package for Social Sciences (SPSS) version 22. The chi-square test was used to test for association with medical wards contamination and wound infection. The quantitative data were analyzed using descriptive statistics, summarized and displayed in tables. The level of significance was set at *P* < 0.05.

### 2.7. Ethical Consideration

The permission to conduct the study was given by the Research Ethics Committee of INES-Ruhengeri Institute of Applied Sciences, and also, an ethical approval letter “Ref 1286/HDR/HRR/2018” was given by Ruhengeri Referral Hospital for sample collection. Patients with wound infection were informed about the study before collecting samples. The right to privacy and confidentiality was respected.

## 3. Results

### 3.1. Demographic Characteristics

Both Gram-positive and Gram-negative bacteria could be isolated in the hospital wards.  Table 1 presents identification of bacteria from three selected hospital wards.

As stated in the table above, Gram-negative bacteria were the most isolates from surgery, pediatric, and intensive care unit with 64.26%, 58.52%, and 29.07%, respectively. Gram-positive isolates stood at 35.19% in surgery, 30.6% in pediatric, and 16.83% in ICU.

### 3.2. Some Items/Working Surface with Reported Contaminating Bacteria in the Hospital Wards

Common bacteria isolated in the surveyed wards are given in Table 2. The prevalence of pathogens among hospital ward's items was 49.1%, 31.1%, 22.9%, 21.3%, and 16.4% for *S. aureus*, *Escherichia coli*, *Pseudomonas aeruginosa*, *Proteus mirabilis*, and *Enterobacter cloacae*, respectively.

### 3.3. Assessment of Airborne Bacterial Biocontamination from Medical Wards

Table 3 provides the list of identified bacteria by air sampling (environmental plate contamination) among studied hospital wards. The predominant environment contaminating bacteria *Pseudomonas aeruginosa* (11.5%) occurred in all assessed medical wards. *Staphylococcus aureus* (9.8%) was the second predominant bacteria and *E. coli* (8.2%) was the third airborne contaminating isolated bacteria.

### 3.4. The Bacteria Isolated in the Wound Sites within Medical Wards

Table 4 provides the percentage of the isolated bacteria in the patients wound site within three medical wards. *S. aureus* (27.9%) was the most isolated bacterium followed by *E. coli* and *P. aeruginosa* at the same of 18.0%, *Proteus mirabilis* was isolated at 11.5%, and *Enterobacter cloacae* was isolated at 4.9%.

### 3.5. Association of Isolated Bacteria with Materials and Wound Sites

According to the table above, the association of medical wards bacterial contamination to wound infection was studied. The bacteria isolates, swabbed materials, and wound sites are given in Table 5. There was a statistical significance association with *E. coli* (*x*^2^ = 10.025, *P*=0.018). Other microorganisms from medical contamination were not statistically significant associated to wound infection.

### 3.6. Antibiotic Susceptibility Patterns of Bacteria Isolate from Wound Sites and Ward Items

The results from Table 6 provide that clindamycin and erythromycin were only effective antibiotics to each of bacteria isolate. *Staphylococcus aureus* was sensitive77.9%, 61.1%, and 55.6% to vancomycin, ciprofloxacin, and cefuroxime, respectively, and resistant to nitrofurantoin 56.6%. *E. coli* isolates were sensitive 77.9%, 55.6%, and 55.6% to cefuroxime, ciprofloxacin, and vancomycin, respectively, and only resistant to nitrofurantoin 44.4%. Vancomycin was effective 100% to *Pseudomonas* and *Proteus* species, and other used antibiotics were effective to them except nitrofurantoin was ineffective 66.7% to *Pseudomonas aeruginosa*. Besides, *Enterobacter* isolates were 100% sensitive to erythromycin, nitrofurantoin, and clindamycin but resistant also 100% to ciprofloxacin, vancomycin, and cefuroxime.

## 4. Discussion

The hospital environment and used equipment play a role in the cross-transmission of multidrug-resistant bacteria, the leading cause of morbidity and mortality in admitted patients globally. This study was conducted to investigate the bacterial contamination of the hospital environment as source of surgical wounds infections. Generally, Gram-negative were predominant bacteria isolates observed compared to Gram-positive isolated bacteria in all sampling sites (Table 1). Gram-negative organisms are responsible for hospital-acquired infections, the *Enterobacteriaceae* family being also the most commonly identified group overall as hospital environment contamination with Gram-positive bacteria results in endemic infections [15]. On the contrary, other studies revealed that contamination with Gram-positive organisms is more widespread than Gram-negative contamination because of the better survival of Gram-positive bacteria in dry air [16]. The finding for general distribution of Gram-negative bacteria isolates was compared to the results from recent studies, the average Gram-negative bacteria count in the operation theatre (OT), intensive care unit, (ICU) and neonatal intensive care unit (NICU) was comprised 45%, 33.9%, and 31%, respectively. Whereas, *Staphylococcus aureus* was observed in general ward and emergency ward of 6 and 5 hospitals (47.18%) [17]. Considering bacterial contamination from ward's items and working surface, results of this research showed that the prevalence of pathogens among hospital ward's items and inanimate surface was 49.1%, 31.1%, 22.9%, 21.3%, and 16.4% for *S. aureus*, *E. coli*, *Pseudomonas aeruginosa*, *Proteus mirabilis*, and *Enterobacter cloacae*, respectively. However, the predominant environment contaminating bacteria *P. aeruginosa* (11.5%) occurred in three assessed medical wards. *Staphylococcus aureus* (9.8%) was the second predominant bacteria, and *E. coli* (8.2%) was the third airborne contaminating isolated bacteria (Tables 2 and 3). In medical wards, inanimate surfaces, higher environment and equipment (e.g., bedrails, stethoscopes, medical charts, and ultrasound machine) may be contaminated by bacteria, including MD isolates. Moreover, cross-transmission of microorganisms from inanimate surface and environmental contamination may have a significant role for hospital-acquired colonization and infections [12, 18]. These findings are similar to those of the Brazilian multicenter study conducted on bacterial contamination of inert hospital surfaces and equipment in critical and noncritical care units that were mostly contaminated not only with *S. aureus* (53.3%) but also with enteric bacteria 30.4%, with a high frequency of samples contaminated with *P. aeruginosa* (64.7%), and *S. aureus* was the main microorganism recovered from the surfaces and equipment [19]. Besides, wound site bacterial infections in the hospital within three medical wards were assessed. *Staphylococcus aureus* (27.9%) was predominant followed by *E. coli*, and *P. aeruginosa* (18.0%), *Proteus mirabilis* (11.5%), and *Enterobacter cloacae* were isolated at 4.9% (Table 4). Consistent with this, several studies have reported that only less than 50% of hospital surfaces are properly cleaned and disinfected with germicides which result to high prevalence of serious infections due to multidrug-resistant pathogens that reached alarming levels in most hospitals [20]. These startling findings strongly suggest that the hospital environment, inanimate surface, and equipment can act as a reservoir of pathogens and enable their cross-transmission to the patient surgical wound site. As well as, contamination may result from healthcare workers' hands or by direct patient shedding of bacteria which are able to survive up to several months on dry surfaces. In regard to the antibiotic susceptibility pattern of bacteria isolates to commonly tested antibiotics in our study, all bacteria isolates were sensitive to clindamycin and erythromycin. The serious resistance of clindamycin and erythromycin was only found for *S. viridans* and *Staphylococcus* in the study conducted from Massachusetts General Hospital [21]. *Staphylococcus aureus* has shown sensitivity to vancomycin, ciprofloxacin, and cefuroxime and resistant to nitrofurantoin. On the other hand, *E. coli* isolates were sensitive to cefuroxime, ciprofloxacin, and vancomycin and resistant also to nitrofurantoin. However, *Pseudomonas* and *Proteus* species were displayed to be 50–100% susceptible to cefuroxime, ciprofloxacin, and vancomycin, which is similar to the study by Goswami et al. [22]. Besides, *Enterobacter* isolates were 100% sensitive to erythromycin, nitrofurantoin, and clindamycin but resistant also 100% to ciprofloxacin, vancomycin, and cefuroxime (Table 5). Considering the commonly used antibiotics in the hospital, it is no longer uncommon to encounter Gram-negative infections that are untreatable using conventional antibiotics in hospitalized patients [23].

## 5. Conclusion

This study was conducted to study the contribution of medical wards contamination to wound infection. Swab specimens were collected for both surface of inanimate object in the hospital wards and patients' wound. However, airborne biocontamination was determined. Culture, Gram stains, biochemical test, and antibiogram test were performed. The medical wards evaluated in this study showed a high bacterial contamination for high-touch inanimate surfaces, hygienic materials, and medical wards environment with bacteria species which are also associated to wound infection. Remarkably, some of bacteria isolates showed resistance to commonly used antibiotics in the hospital. Healthcare professionals should be aware that medical wards environment, surface of inanimate object, and patient's hygienic materials can contain bacterial contaminants, which can contribute to wound infection and other hospital-acquired infections. Therefore, the periodic hospital wards decontamination is highly recommended. Further studies are recommended to use an impaction method to determine hospital environment biocontamination.

## Figures and Tables

**Table 1:** Numbers and frequency of bacteria isolates in the different wards of hospital.

	Types of specimens collected and number of isolates				
Surveyed clinics	Airborne sampling by agar plates sedimentation	Patients' wound swabs	Surface samples swabs (blanket, door, backet)	Isolated G^+ve^ bacteria	Isolated G^−ve^ bacteria
Surgery	4	10	11	23 (35.19)	42 (64.26)
ICU	4	7	5	11 (16.83)	19 (29.07)
Pediatric	4	6	10	20 (30.6)	38 (58.52)

**Total isolates**				153	

**Table 2:** Bacterial contamination from clinic items and working surface.

Level and type of bacteria isolates					
Clinic and surface sample swabs	*Escherichia coli*	*Pseudomonas aeruginosa*	*Proteus mirabilis*	*S. aureus*	*Enterobacter cloacae*
ICU					
Hygienic materials	1 (1.6)	0	0	1 (1.6)	0
Wall near patients	0	1 (1.6)	1 (1.6)	3 (4.9)	1 (1.6)
The door	1 (1.6)	1 (1.6)	2 (3.3)	2 (3.3)	1 (1.6)
Blanket/bed sheet	1 (1.6)	0	0	1 (1.6)	0
Pediatric					
Hygienic material	3 (4.9)	1 (1.6)	1 (1.6)	3 (4.9)	1 (1.6)
Wall near patients	1 (1.6)	2 (3.3)	2 (3.3)	4 (6.5)	2 (3.3)
Doors	2 (3.3)	1 (1.6)	1 (1.6)	1 (1.6)	1 (1.6)
Blanket/bed sheets	3 (4.9)	1 (1.6)	2 (3.3)	3 (4.9)	1 (1.6)
Surgery					
Hygienic materials	1 (1.6)	2 (3.3)	0	3 (4.9)	1 (1.6)
Wall near patients	2 (3.3)	2 (3.3)	2 (3.3)	4 (6.5)	1 (1.6)
Blanket/bed sheets	3 (4.9)	2 (3.3)	2 (3.3)	3 (4.9)	0
Doors	1 (1.6)	1 (1.6)	0	2 (3.3)	1 (1.6)
Total	19 (31.1%)	14 (22.9%)	13 (21.3%)	30 (49.1%)	10 (16.4%)

**Table 3:** Bacteriological identification by wards with plates settling biocontamination.

Bacteria isolates			
Clinics	*E. coli*	*P. aeruginosa*	*S. aureus*
ICU	0	2 (3.3)	1 (1.6)
Pediatric	3 (4.9)	2 (3.3)	3 (4.9)
Surgery	2 (3.3)	3 (4.9)	3 (4.9)
Total	5 (8.2)	7 (11.5)	6 (9.8)

**Table 4:** Bacteriological contamination in the patient wounds.

Level and frequency of bacteria isolates					
Wards	*E. coli*	*P. aeruginosa*	*Proteus mirabilis*	*S. aureus*	*Enterobacter cloacae*
ICU	2 (3.3)	3 (4.9)	2 (3.3)	3 (4.9)	0
Pediatric	4 (6.5)	3 (4.9)	2 (3.3)	6 (9.8)	0
Surgery	5 (8.2)	5 (8.2)	3 (4.9)	8 (13.1)	3 (4.9)
Total	11 (18.0)	11 (18.0)	7 (11.5)	17 (27.9)	3 (4.9)

**Table 5:** Bacteria isolates and their association with swabbed materials and wound sites.

Types of bacteria isolated	Swabbed materials and sites					Chi-square		
	Status	Bed sheet	Hygienic materials	The door	Wall near patients	*X* ^2^	df	*Pvalue*
*E. coli*	Negative	0	3	2	8	10.025	3	0.018
	Positive	7	5	5	3			
*P. aeruginosa*	Negative	4	5	4	6	0.122	3	0.989
	Positive	3	3	3	5			
*Proteus* spp.	Negative	4	6	4	6	0.934	3	0.817
	Positive	3	2	3	5			
*S. aureus*	Negative	0	1	2	1	2.84	3	0.417
	Positive	7	7	5	10			
*Enterobacter*	Negative	6	5	5	9	1.435	3	0.697
	Positive	1	3	2	2			

Pearson chi-square = 10.025; Df = 3; *P*=0.018.

**Table 6:** Sensitivity testing of used antibiotic discs.

Isolates	*E. coli*		*P. aeruginosa*		*Proteus mirabilis*		*Enterobacter cloacae*		*S. aureus*	
Antibiotic	S	R	S	R	S	R	S	R	S	R
E	55.6	44.4	66.7	33.3	50	50	100	0	55.6	44.4
CIP	55.6	44.4	66.7	33.3	100	0	0	100	61.1	38.9
F	44.4	55.6	33.3	66.7	50	50	100	0	44.4	56.6
VA	55.6	44.4	100	0	100	0	0	100	77.9	22.1
CXM	77.9	22.1	76.7	23.3	50	50	0	100	55.6	44.4
DA	66.7	33.3	68.7	21.3	100	0	100	0	66.7	33.3

E, erythromycin; CIP, ciprofloxacin; F, nitrofurantoin; VA, vancomycin; CXM, cefuroxime; DA, clindamycin.

## Data Availability

The data used to support the results of this study are available from the corresponding author upon request.
